# The Response of Human Macrophages to β-Glucans Depends on the Inflammatory Milieu

**DOI:** 10.1371/journal.pone.0062016

**Published:** 2013-04-24

**Authors:** Cristina Municio, Yolanda Alvarez, Olimpio Montero, Etzel Hugo, Mario Rodríguez, Esther Domingo, Sara Alonso, Nieves Fernández, Mariano Sánchez Crespo

**Affiliations:** 1 Instituto de Biología y Genética Molecular, CSIC, Valladolid, Spain; 2 Centro para el Desarrollo de la Biotecnología, CSIC, Parque Tecnológico de Boecillo, Valladolid, Spain; 3 Departamento de Bioquímica y Biología Molecular, y Fisiología, Universidad de Valladolid, Valladolid, Spain; Universidad Pablo de Olavide, Centro Andaluz de Biología del Desarrollo-CSIC, Spain

## Abstract

**Background:**

β-glucans are fungal cell wall components that bind to the C-type lectin-like receptor dectin-1. Polymorphisms of dectin-1 gene are associated with susceptibility to invasive fungal infection and medically refractory ulcerative colitis. The purpose of this study has been addressing the response of human macrophages to β-glucans under different conditions mimicking the composition of the inflammatory milieu in view of the wide plasticity and large range of phenotypical changes showed by these cells, and the relevant role of dectin-1 in several pathophysiological conditions.

**Principal Findings:**

Serum-differentiated macrophages stimulated with β-glucans showed a low production of TNFα and IL-1β, a high production of IL-6 and IL-23, and a delayed induction of cyclooxygenase-2 and PGE_2_ biosynthesis that resembled the responses elicited by crystals and those produced when phagosomal degradation of the phagocytic cargo increases ligand access to intracellular pattern recognition receptors. Priming with a low concentration of LPS produced a rapid induction of cyclooxygenase-2 and a synergistic release of PGE_2_. When the differentiation of the macrophages was carried out in the presence of M-CSF, an increased expression of dectin-1 B isoform was observed. In addition, this treatment made the cells capable to release arachidonic acid in response to β-glucan.

**Conclusions:**

These results indicate that the macrophage response to fungal β-glucans is strongly influenced by cytokines and microbial-derived factors that are usual components of the inflammatory milieu. These responses can be sorted into three main patterns i) an elementary response dependent on phagosomal processing of pathogen-associated molecular patterns and/or receptor-independent, direct membrane binding linked to the immunoreceptor tyrosine-based activation motif-bearing transmembrane adaptor DNAX-activating protein 12, ii) a response primed by TLR4-dependent signals, and iii) a response dependent on M-CSF and dectin-1 B isoform expression that mainly signals through the dectin-1 B/spleen tyrosine kinase/cytosolic phospholipase A_2_ route.

## Introduction

Classical distinction between macrophage types includes the type M1 inflammatory macrophage and the M2 regulatory macrophage. In cultures starting from peripheral blood monocytes, supplementation with human serum, a source of M-CSF but not GM-CSF [1], produces differentiated but non-polarized macrophages (M0 type), and addition of different cytokine cocktails elicit polarization versus the M1 or M2 type. One of the most used macrophage stimuli are zymosan particles. These contain β-glucans and α-mannans, and activate macrophages through different receptors, among which dectin-1 (encoded by the *clec7a* gene) is the most important receptor for β-glucans [2]. The response of macrophages to β-glucans has mainly been studied in rodents and few reports have been conducted in human macrophages, even though dectin-1 plays a relevant role in human disease since polymorphisms of *clec7a* are associated with an increased risk of fungal infection [3] and medically refractory ulcerative colitis [4]. The expression of dectin-1 is widely distributed in myeloid cells and is modulated by cytokines and microbial products [5], [6]. Transcription from human *clec7a* gives rise to the expression of several isoforms of the receptor [5], but a survey of dectin-1 expression along the differentiation of human macrophages has not been conducted yet. This is of pathophysiological relevance since monocytes can display different responses to β-glucans [7], [8]. Systematic studies in mouse macrophages have disclosed that the response to β-glucans depends on the activation state of the cell and, in general, BMDM are poor responders in spite of the expression of dectin-1, thus making the response dependent on myeloid cell programming [9]. Whereas GM-CSF and IFNγ allow for the release of TNFα in response to β-glucans, mouse macrophages differentiated with M-CSF did not produce cytokines [10]. Although a complete mechanistic explanation for these findings is not available, this points to a cell-type specific variability of CARD9-mediated NF-κB activation downstream of dectin-1 ligation, although other dectin-1 associated signals are preserved. Dectin-1 cooperates with other receptors, for instance, CR3 [11], TLR2 [12], [13], DC-specific ICAM-3 grabbing nonintegrin (DC-SIGN) [14], and galectin-3 [15]. Cooperation of dectin-1 and TLR2 is of particular relevance since the sole stimulation of mouse thioglycollate-elicited macrophages with purified β-glucans fails to induce cytokine production even though spleen tyrosine kinase (Syk) activation occurs, whereas combined stimulation of dectin-1 and TLR2 elicits a robust response in a MyD88 and Syk-dependent manner [16]. The case of CR3 is also relevant since it may engage β-glucans through its C-lectin-like domain and it also binds β-glucan particles that have been opsonized with CR3 through the I domain. In fact, opsonisation of zymosan is widely used to induce productive binding in polymorphonuclears and monocytes to study the release of lipid mediators [8], [17] and complement is of central importance for neutrophil response to *Candida* infection [18]. CR3 signaling involves the recruitment of adaptors containing immunoreceptor tyrosine-based activation motifs (ITAM) such as DNAX-activating protein (DAP)12 and Fc receptor γ-chain, and the tyrosine kinase Syk [19]. Notably, this mechanism mimics dectin-1 signaling that depends on its own ITAM.

A new scenario has emerged after disclosing the involvement of PGE_2_ in the induction of Th2 type immune responses by particulate crystal-like materials [20]. In fact, alum and silica induce the production of PGE_2_ through a receptor-independent mechanism triggered from phagolysosomes that leads to the production of antigen-specific serum IgE. This mechanism is also triggered by sodium monourate particles [21] and hemozoin crystals [22], and mimics the reported activation of Syk by receptor-independent, direct membrane binding of monosodium urate crystals in dendritic cells (DC) [23]. In addition to the significance of these findings to vaccine technology, these results are relevant to understand the pathogenic mechanism underlying the inflammation induced by microcrystals in gouty arthritis and silicosis [24] and highlight the complexity of the process of recognition of particles bearing pathogen-associated molecular patterns (PAMP), which may include receptor-dependent and independent mechanisms, and is influenced by receptor expression, the state of differentiation of the cell, and the presence of molecules that affect receptor function.

In this study, we have observed that stimulation of putative M0 macrophages with zymosan induces COX-2 expression, release of PGE_2_, IL-6, and IL-23, and activation of the NF-κB system in a way that suggests the involvement of phagosomal processing and may be primed by low concentrations of LPS and interferons. Differentiation of monocytes with M-CSF produced an increase of dectin-1 B isoform expression, an increased capacity to release arachidonic acid (AA), and a parallel decrease of the production of IL-6 and IL-23. These results disclose three different patterns of response to zymosan, the features of which are: i) a response consistent with phagosomal processing, ii) a response primed by LPS and cytokines, and iii) a response associated with an enhancement of dectin-1 B isoform expression by M-CSF that mainly conveys signals through the dectin-1/Syk/cPLA_2_ route.

## Materials and Methods

### Ethic Statement

The study was approved by the Bioethical Committee of the Spanish Council of Research (CSIC) and the written informed consent of all healthy donor subjects was obtained at Centro de Hemoterapia y Hemodonación de Castilla y León Biobank. The participants received written consent according to the regulations of the Biobank. The researchers received the samples in an anonimous way. The process is documented by the Biobank authority according to the specific Spanish regulations. The ethics committee approved this procedure before starting the study.

### Cells and Reagents

Monocytes were isolated from pooled buffy coats of healthy volunteer donors. Differentiation of monocytes into macrophages was carried out by culture in the presence of 5% human serum for seven days or in 10% fetal bovine serum, supplemented with 10 ng/ml M-CSF. Human monocyte-derived dendritic cells (DC) were obtained by culture in the presence of GM-CSF (800 U/ml) and IL-4 (500 U/ml) for 5 days and differentiation assessed by immunofluorescence flow cytometry of CD40, CD80, CD83 and CD86 as reported [14]. Mouse macrophages were obtained from the bone-marrow of *dectin-1^−/−^* and wild type (WT) animals in a CB57/BL6 background [25] by differentiation in the presence of murine M-CSF. Zymosan from *Saccharomyces cerevisiae*, mannan, laminarin, latex beads, and LPS were from Sigma Chemical Co. (St. Louis, MO). Depleted zymosan was from InvivoGen (San Diego, CA). Pure β-glucan was obtained from Dr. David L. Williams from East Tennessee State University. Endotoxin levels in the reagents were below 1 ng/ml as determined by the *Limulus* Amebocyte Lisate assay (Cambrex Bio Sciences, Walkersville, MD). Moreover, addition of 200 µg/ml polymyxin B did not modify the effect of stimuli other than LPS, what negates the possible involvement of LPS in the responses to β-glucans.

Coating of C3bi to zymosan was conducted by incubation with human serum. TNFα, IL-1β, IL-23, and IL-6 were assayed by ELISA. The expression of the different isoforms of dectin-1 was carried out by RT-PCR and the PCR products identified by DNA sequencing on both strands. The sequences of the primers are shown in Table 1.

**Table 1:** Oligonucleotide primers used for real time RT-PCR and ChIP.

Gene	Sequence (5′-3′)	GeneBank AC
*clec7a* (dectin-1) exon 1 forward	GGGCTCTCAAGAACAATGGA	AF400596
exon 6 reverse	TTGGAGATGGGTTTTCTTGG	
exon 5 reverse	CCCAGAGCCATGGTACCT	
*CD209* (DC-SIGN) forward	AGGTCCCCAGCTCCATAAGT	NM_021155
reverse	TCTCTGGAAGCTCACCCACT	
*tlr2* (TLR2) forward	GCCAAAGTCTTGATTGATTGG	NM_003264
reverse	TTGAAGTTCTCCAGCTCCTG	
*mrc1* forward	GCTGAACCTGGAAAAAGCTG	NM_002438
reverse	ACGAAGCCATTTGGTAAACG	
*gapdh* forward	GTCAGTGGTGGACCTGACCT	NM_002046.3
reverse	AGGGGAGATTCAGTGTGGTG	
*ptgs2* promoter forward	AGGAGAGGGAGGGATCAGAC	D28235
reverse	TTTACCCACGGAAATGAGAAA	

*mrc1* stands for the mannose receptor gene.

### AA Release Assay and Prostaglandin E_2_ (PGE_2_) Production

Radioactive labeling with [^3^H]AA was performed by overnight incubation in the presence of 0.25 µCi/ml [^3^H]AA in 0.25% essentially fatty acid-free BSA. After labeling, cells were washed and equilibrated at 37°C in medium containing 1% BSA, before the addition of agonists or vehicle. The release of [^3^H]AA into the culture medium after 60 min stimulation was measured by scintillation counting. PGE_2_ was assayed with Biotrack ELISA system. The characterization of PGE_2_ was carried out by reversed phase ultra performance liquid chromatography and electrospray-quadrupole-time-of-flight-mass spectrometry (Materials S1).

### Assays for Phagocytosis and Flow Cytometry

For the assay of phagocytosis, cells were incubated with zymosan particles conjugated with Alexa Fluor® 488 at the concentration of five particles per cell at 37°C for the times indicated. Subsequently, cells were washed, treated with 100 units/ml lyticase in phosphate-buffered saline (PBS) for 10 min at room temperature in order to dissolve extracellular zymosan, and then resuspended in 500 µl of PBS supplemented with 0.05% sodium azide and 1 mM EDTA for analysis by flow cytometry [7]. For the assay of the expression of receptors on the cell membranes, adherent cells were scraped and centrifuged for 5 min at 350×g and resuspended in PBS. Ab was added at the concentration of 0.5 µg for 5.10^5^ cells and incubated for 45 min at 4°C. When the Ab were labelled with FITC, cells were washed and fixed in 1% formaldehyde. In the case of non labelled Ab, indirect immunofluorescence was carried out using a FITC-labeled secondary Ab, before washing and formaldehyde fixation steps. Isotype-matched irrelevant Ab were used as control. The analysis was performed in a Gallios Flow Cytometer (Becton Dickinson). At least 10,000 cells were analyzed per sample. Kaluza software version 1.1 and FlowJo software were used for quantitative data analysis and preparation of overlay histograms.

### Immunoblots and Immunoprecipitations

Samples were loaded on a 10% SDS-PAGE system. Proteins were transferred to nitrocellulose membranes and the membranes used for immunodetection of cyclooxygenase-2 (COX-2) (Santa Cruz sc-1745, Ab), cytosolic phospholipase A_2_ (cPLA_2_), (Cell Signaling # 2832, Ab) and phospho-Syk (Santa Cruz sc-2711, Ab) using the Amersham Biosciences ECL system. β-actin and TATA-box binding protein (TBP) were used as a load control. For the assay of nuclear proteins, the nuclear extracts were obtained with an extraction kit (Active Motif). The membranes were used for the detection of RelA/p65, c-Rel, and p50. Immunoprecipitations were carried out as described [14] using anti-DAP12 Ab (Santa Cruz sc-20783), anti-CD32A Ab (abcam ab41899), and anti-Fc receptor γ-chain Ab (Millipore # 06-727) Ab. Briefly, cells were lysed in a medium containing 20 mM Tris-HCl (pH 7.5), 150 mM NaCl, 5 mM EDTA, 1% Nonidet P-40, 1 mM Na_3_VO_4_, 10 µg/ml aprotinin and leupeptin, 100 µg/ml soybean trypsin inhibitor, and 1 mM PMSF and clarified by centrifugation at 15,000 rpm for 20 min. The clarified lysates were preabsorbed on protein G-Sepharose and then incubated with precipitating mAb for 4 hours, followed by overnight incubation with protein G-Sepharose beads. Immune complexes were extensively washed, suspended in Laemmli sample buffer and subjected to SDS/PAGE. Blots were stained to assess the tyrosine phosphorylation of FcR-γ-chain, DAP12, and CD32A with anti-phosphotyrosine, clone 4G10 (Millipore # 05-321) mAb.

### Cromatin Immunoprecipitation (ChIP) Assay

ChIP assays were conducted with reagents and Ab against c-Rel (Santa Cruz sc-70), RelA/p65 (# 06-418), and Ac-14K-histone H3 (# 07-353) from Upstate Biotechnology as previously reported [26]. Briefly, cells were stimulated and then washed twice with PBS and fixed with 1% formaldehyde. Cross-linking was terminated by 0.125 M glycine. Crude nuclear extracts were collected by microcentrifugation and resuspended in a lysis buffer containing a high salt concentration. Chromatin sonication was carried out using a Bioruptor device from Diagenode (Diagenode, Liege, Belgium). The chromatin solution was precleared by adding Protein A/G PLUS-Agarose for 30 min at 4°C under continuous rotation. After elimination of the beads, Ab was added for overnight incubation al 4°C, and then Protein A/G PLUS-Agarose was added and incubated for an additional period of 2 hours at 4°C. Beads were harvested by centrifugation at 12,000 rpm and sequentially washed with lysis buffer high salt, wash buffer, and elution buffer. Cross-links were reversed by heating at 67°C in a water bath, and the DNA bound to the beads isolated by extraction with phenol/chloroform/isoamylalcohol. PCR reactions were carried out with primers designed from the *pgts2* promoter (Table 1).

### Real-time RT-PCR

Purified RNA was used for RT reactions. The resulting cDNA was amplified in a PTC-200 apparatus equipped with a Chromo4 detector (BioRad) using SYBR Green I mix containing HotStart polymerase (ABgene). Cycling conditions were adapted to each set of primers. *gadph* was used as a housekeeping gene to assess the relative abundance of the different mRNA, using the comparative C_T_ (circle threshold) method.

### Statistical Analysis

Data are represented as the mean ± S.D. and were analyzed with the Prism 4.0 statistical program (GraphPad Software). Comparison between two experimental groups was carried out using the Student *t* test. Differences were considered significant for *p*<0.05.

## Results

### Particle Uptake and AA Release

**Figure 1:**
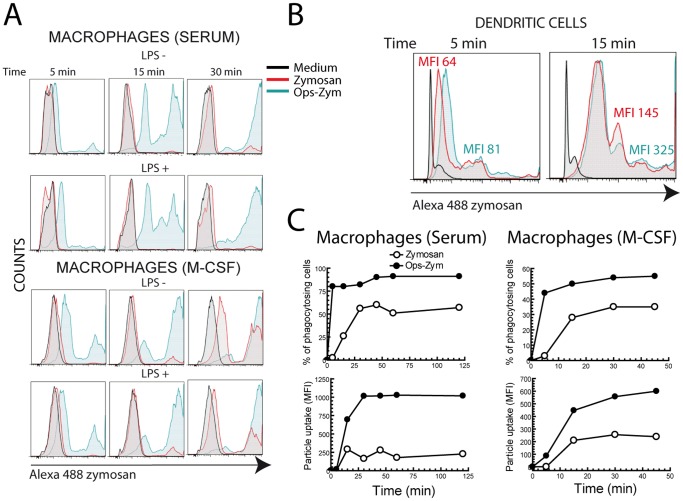
Zymosan uptake by macrophages and DC. (A, B and C) Macrophages and DC were incubated with Alexa Fluor® 488-labeled zymosan at the concentration of 5 particles per cell at 37°C for the times indicated and the uptake of particles assayed by flow cytometry. Results in (A) show representative experiments in macrophages differentiated in the presence of human serum or M-CSF for seven days and then treated for 3 hours with 10 ng/ml LPS or left untreated prior to the addition of zymosan particles. (B) Zymosan uptake by DC. Experiments in (C) were conducted to compare percentage of phagocytosing cells and MFI in serum and M-CSF differentiated macrophages. Ops-Zym indicates serum opsonised zymosan. Results show a representative experiment of three independent ones with a similar trend.

**Figure 2:**
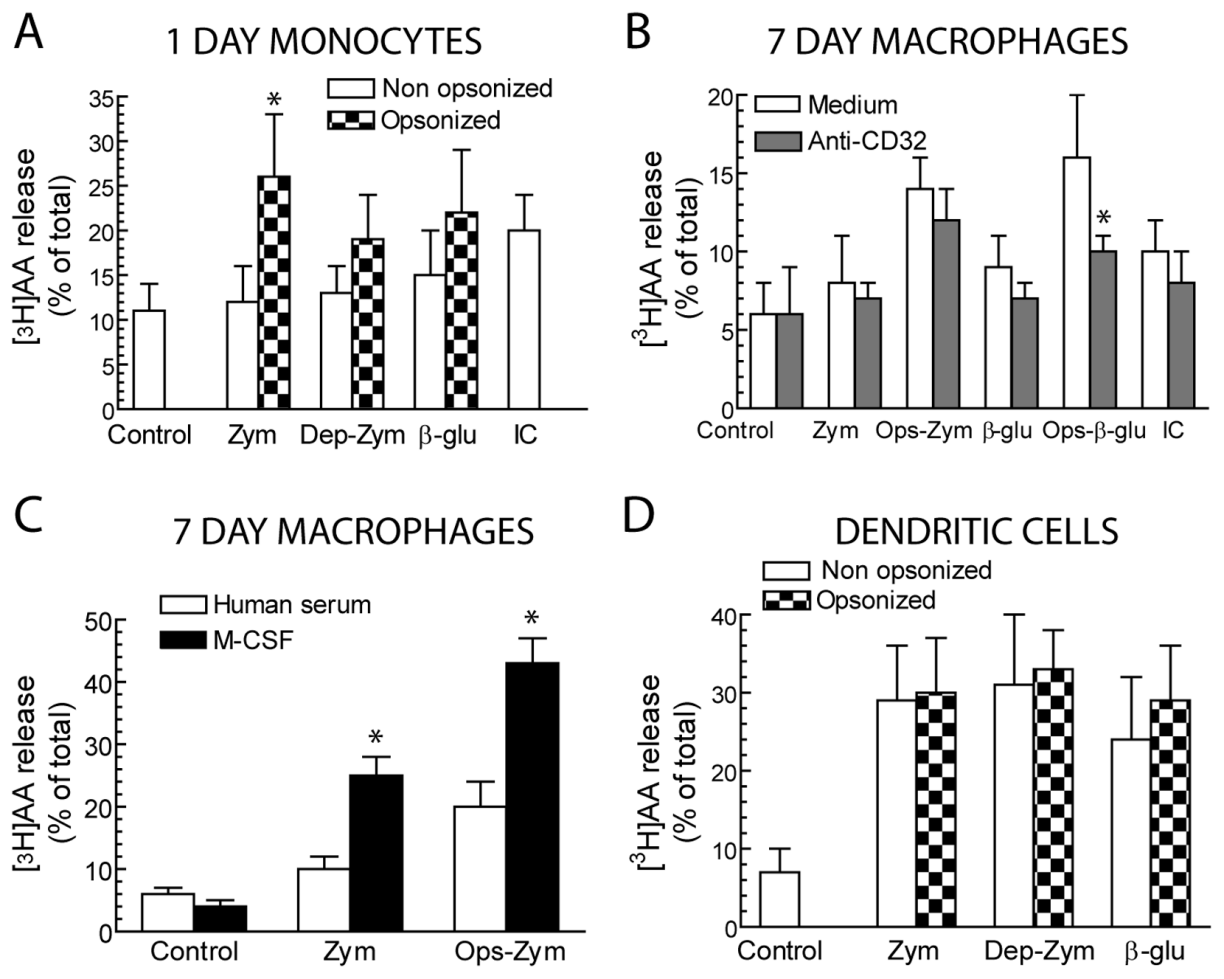
Release of [^3^H]AA. Cells were differentiated and stimulated as indicated. Anti-CD32A antibody was used at the concentration of 10 µg/ml 30 min prior to the addition of the stimuli. Zymosan (Zym), Ops-Zym, depleted zymosan (Dep-Zym), and β-glucan (β-glu) particles were used at the concentration of 1 mg/ml. Immune complexes (IC) were used at the concentration of 100 µg/ml. Results represent mean ± S.D. of 5 to 6 independent experiments. *Indicates *p*<0.05.

Monocyte-derived macrophages differentiated with serum showed a limited uptake of zymosan particles (Figure 1A). Opsonisation of the particles with fresh serum increased the uptake to the levels observed in DC, which phagocytose both zymosan and opsonized zymosan efficiently (Figure 1B and 1C). The uptake of opsonized zymosan reached maximal levels at ∼30 min as assessed by both the percentage of cells showing phagocytosis and the increase of mean fluorescence intensity (MFI), which is an indicator of the number of particles taken up by each cell. Particle uptake was not affected by priming with 10 ng/ml LPS (Figure 1A) and only slightly by differentiation with M-CSF (Figure 1B, lower panels, and 1C, right panels). Stimulation of monocytes and serum-differentiated macrophages with zymosan, depleted zymosan, and pure β-glucan particles induced a negligible release of [^3^H]AA (Figure 2A and B). In contrast, zymosan produced a significant release of [^3^H]AA in both macrophages differentiated with M-CSF and in DC (Figure 2C and D). Notably, whereas zymosan opsonisation did not significantly increase the ability of zymosan to release [^3^H]AA in DC, opsonisation did increase [^3^H]AA release in macrophages (Figure 2A–D).

### PGE_2_ Production and COX-2 Induction

**Figure 3:**
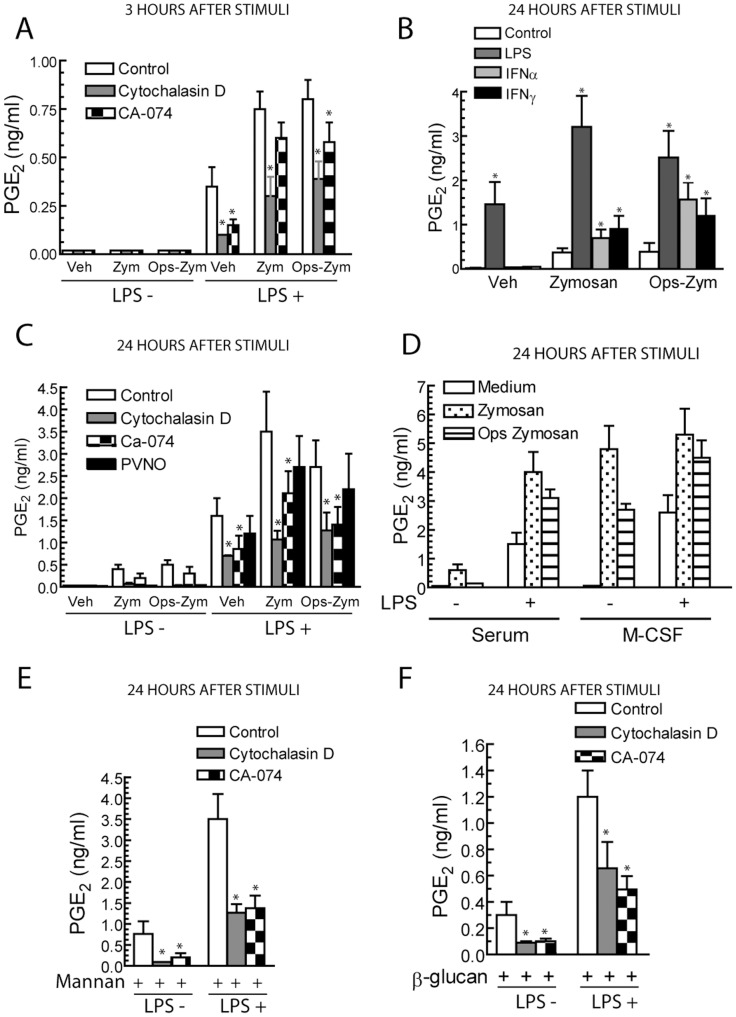
Production of PGE_2_. (A, C, E and F) Macrophages were primed with 10 ng/ml LPS or (B) 100 U/ml IFNα and IFNγ for 3 hours or left untreated, and then stimulated with different additions at a concentration of 1 mg/ml. 10 µM CA-074, 2 µM cytochalasin D, and 20 µg/ml PVNO were added before the stimuli. The supernatants were collected for the assay of PGE_2_. Results represent mean ± S.D. of 6 to 7 independent experiments. Veh, indicates vehicle, *indicates *p*<0.05.

**Figure 4:**
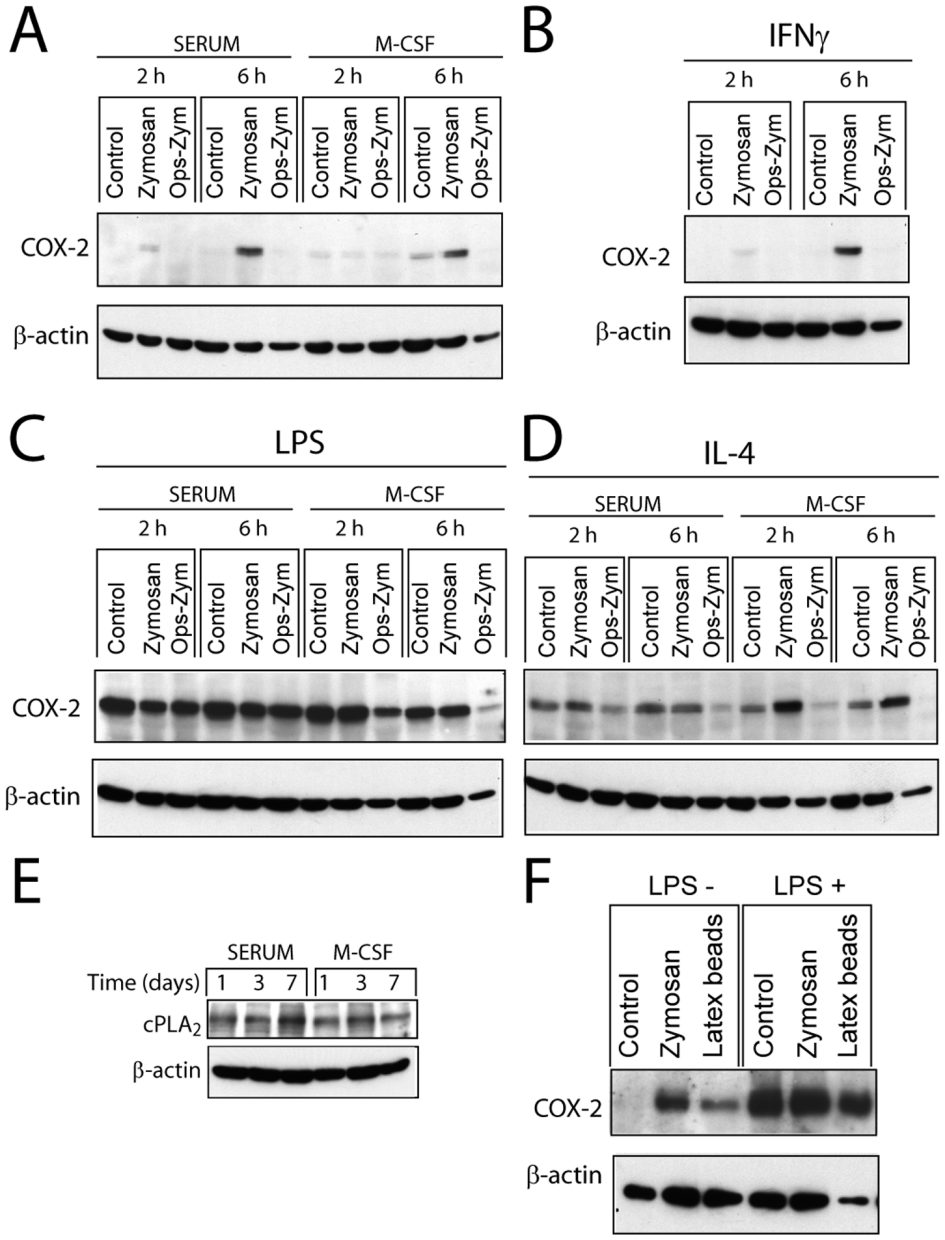
Expression of COX-2 protein. **(**A–F**)** Macrophages were differentiated in the presence and absence of M-CSF and then primed with (B) 100 U/ml IFNγ, (C and F) 10 ng/ml LPS, and (D) 500 U/ml IL-4 for 3 hours. At the times indicated after stimulation, cell lysates were collected for the immunodetection of COX-2 and cPLA_2_ proteins. These are representative of experiments conducted at least in duplicate. (E) The expression of cPLA_2_ was assayed in macrophages differentiated in the presence of serum and M-CSF. (F) Macrophages were stimulated with latex beads at a concentration of 60 particles per cell.

Stimulation of serum-differentiated macrophages with zymosan for 3 hours did not induce the production of PGE_2_, but concentrations of ∼0.5 ng/ml were detected at 24 hours. Priming with LPS increased the production of PGE_2_ (Figure 3A–C), thus suggesting that low concentrations of LPS elicit a synergistic effect. Priming with IFNα and IFNγ also increased the production of PGE_2_ (Figure 3B). In the case of macrophages differentiated with M-CSF, the production of PGE_2_ was enhanced (Figure 3D), but it did not show further significant increase by LPS priming. Since zymosan contains both α-mannan and β-glucan polymers, additional experiments were carried out with mannan and β-glucan. LPS showed an enhancing effect similar to that observed with zymosan particles (Figure 3E and F). Altogether, these results indicate that the response to zymosan is synergistically enhanced by stimuli used to generate type M1 polarized macrophages and by M-CSF. Since the production of PGE_2_ depends on the activity of two enzymes: the constitutively expressed COX-1 and the inducible COX-2, the expression of COX-2 was addressed. As shown in Figure 4A and B, zymosan induced the expression of COX-2 at 6 hours. Unlike M-CSF and IFNγ, priming with LPS induced a strong induction of COX-2 protein (Figure 4C). IL-4, which is used to induce macrophage M2 type differentiation elicited a slight induction of COX-2 at 2 hours (Figure 4D). Differentiation with M-CSF did not influence the expression of cPLA_2_ (Figure 4E). To confirm that products of the COX routes were the predominant eicosanoids released, mass spectrometric analysis was conducted. Stimulation with zymosan of LPS-primed macrophages induced a predominant production of PGE_2_, a lower production of PGD_2,_ and a minimal amount of leukotriene (LT) B_4_ (Table 2 and Figure S1). Notably, in cells differentiated with M-CSF, there was a detectable production of LTB_4_ that agrees with the increased release of AA upon zymosan challenge and processing via the 5-lipoxygenase route. These data indicate that priming with LPS, IFN, and M-CSF increases the production of PGE_2_.

**Table 2:** Eicosanoid release by macrophages.

**Differentiation**	**Treatment**	**PGE_2_**	**PGD_2_**	**LTB_4_**
Human serum	None	0 ng/ml	0.01 ng/ml	0 ng/ml
Human serum	LPS+Zymosan	7 ng/ml	1 ng/ml	0.02 ng/ml
M-CSF	None	0 ng/ml	0 ng/ml	0 ng/ml
M-CSF	LPS+Zymosan	6.9 ng/ml	1.2 ng/ml	0.2 ng/ml

Human macrophages were primed for 3 hours with LPS and stimulated for 24 hours with zymosan. Results represent mean values of one experiment with duplicate samples.

### Cytokine Production

**Figure 5:**
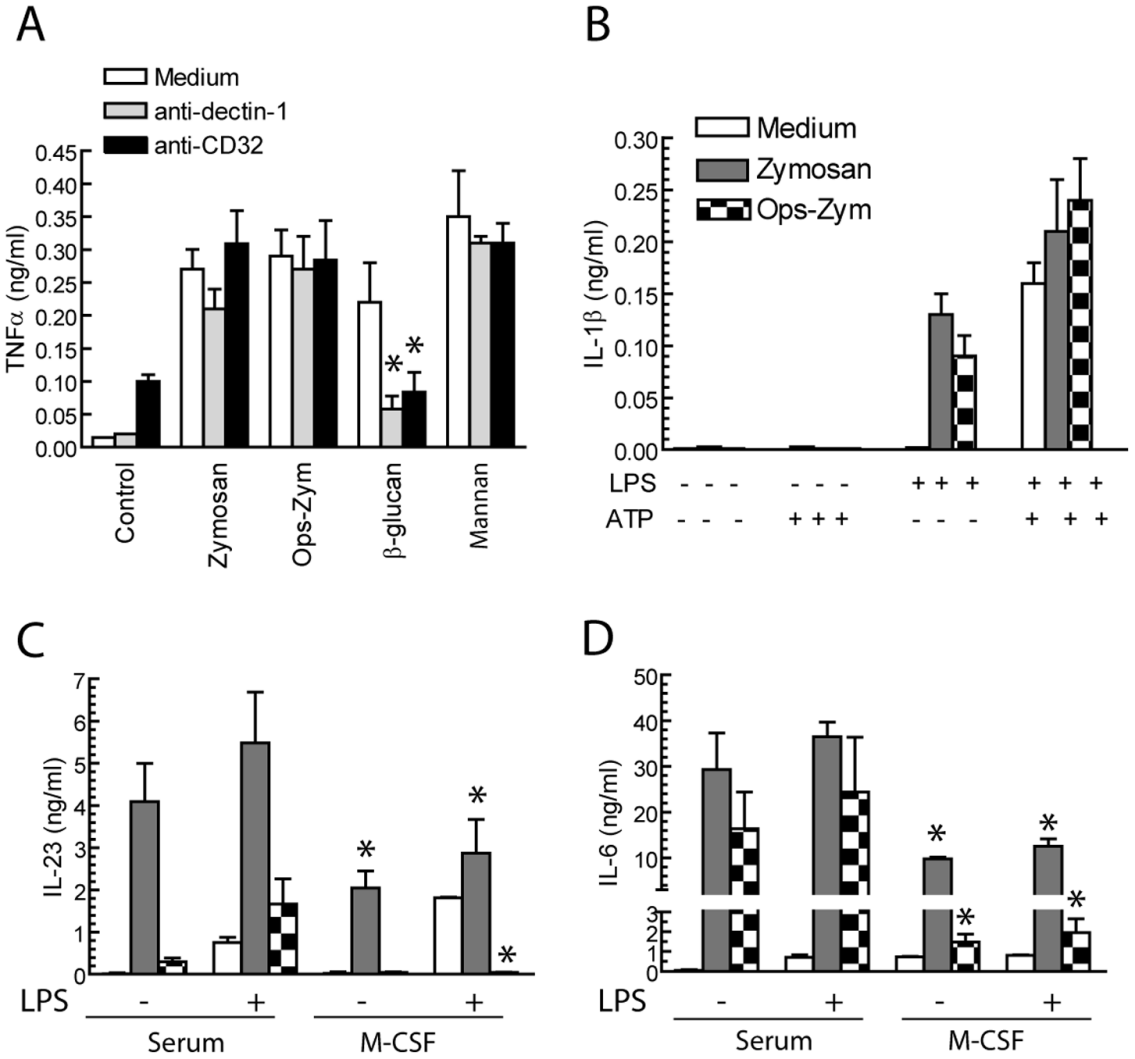
Zymosan uptake by macrophages and DC.Assay of cytokine production. Macrophages were stimulated as indicated and after 24 hours the supernatants were collected for the assay of cytokines. (A) Inhibitory anti-CD32 and anti-dectin-1 Ab were used at a concentration of 10 µg/ml, 30 min prior to the addition of the stimuli. (B) The combination of a priming concentration of LPS and 10 mM ATP was used as a control of inflammasome activation. Results represent mean ± S.D. of 4 independent experiments in the case of TNFα (A) and IL-1β (B), and three experiments in the case of IL-23 (C) and IL-6 (D). *Indicates *p*<0.05. In (C) and (D) comparison has been conducted between serum and M-CSF differentiated macrophages.

Zymosan, which is a potent stimulus for the release of cytokines in many cell systems [27]–[29], induced a low amount of TNFα (Figure 5A), what differs from findings in DC where it behaves as a strong stimulus [28]. In the case of β-glucan particles, the production of TNFα was inhibited by blocking Ab against dectin-1 and CD32A. Zymosan released low amounts of IL-1β, even in the presence of 10 ng/ml LPS, and this effect was increased in the presence of 10 mM ATP (Figure 5B). In contrast, zymosan was a potent inducer of IL-6 and IL-23 production (Figure 5C and D). Unlike what was observed on AA release and PGE_2_ production, differentiation with M-CSF blunted the release of the cytokines (Figure 5D). These data indicate that zymosan is a robust stimulus for IL-6 and IL-23 production and that this production is modulated by the inflammatory environment.

### Expression of the Receptors Involved in Zymosan Recognition

**Figure 6:**
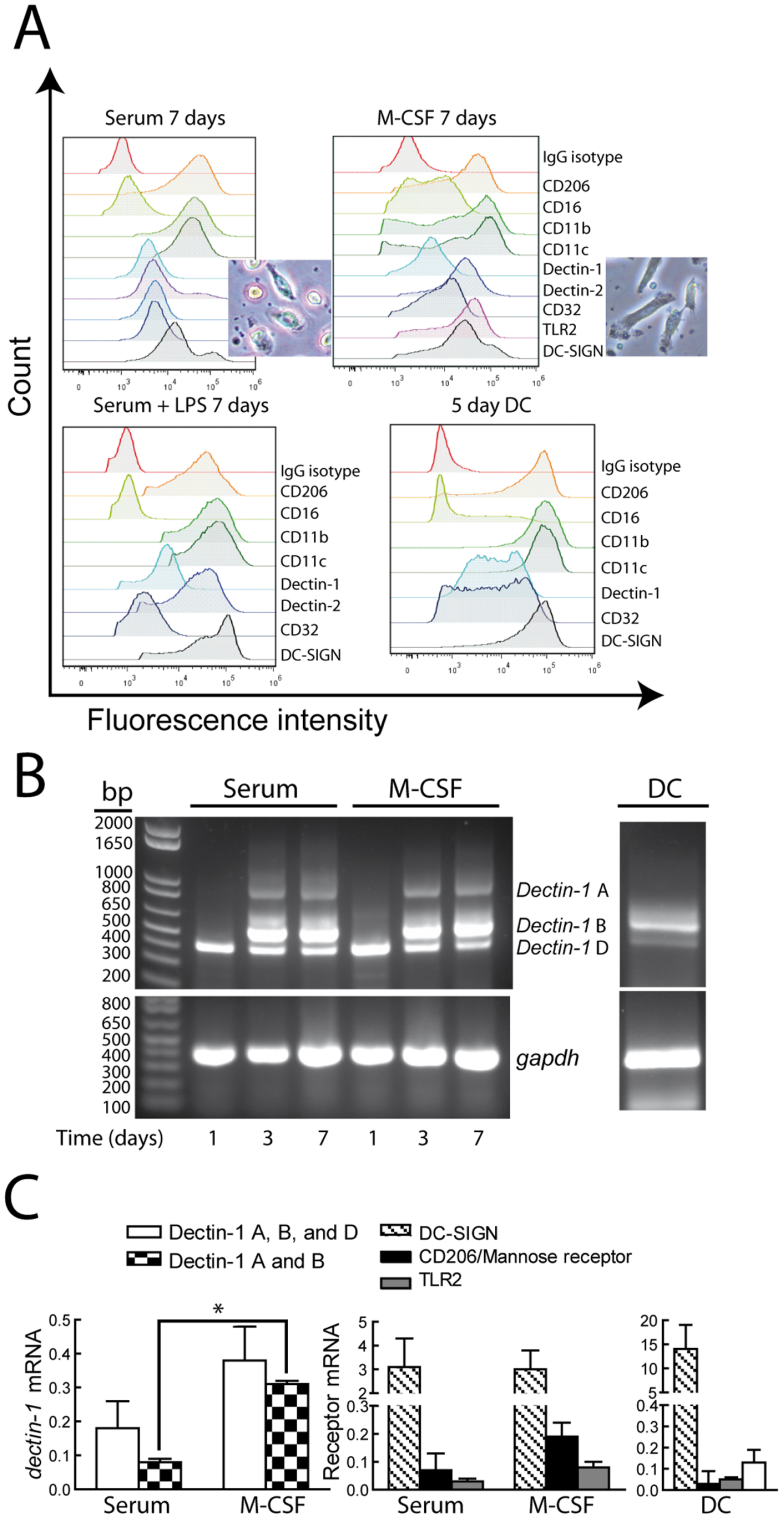
Expression of receptors. (A) The expression of different receptors was assayed by flow cytometry. The panel represents a typical experiment of two. (B) Expression of dectin-1 mRNA. The image shows a 40 cycles PCR carried out to show the minor band corresponding to dectin-1 A isoform that was not observed with a lower number of cycles. The high number of PCR cycles explains the difficulty to assess in the image the increased expression of dectin-1 B isoform in M-CSF differentiated macrophages over serum-differentiated macrophages that could be detected in real-time RT-PCR. The identification of the different isoforms was carried out by DNA sequencing on both strands of the RT-PCR product. This is a representative experiment of two. (C) Real-time RT-PCR was carried out with reverse primers in exon 5 and exon 6 to assay dectin-1 A and B isoforms, as well as with primers to assay DC-SIGN, the mannose receptor, and TLR2 in 7 day differentiated macrophages and DC. *gadph* was assayed as a load control and as a reference in real-time RT-PCR. Results represent mean ± S.D. of 5 experiments. *Indicates *p*<0.05.

A plausible explanation for the different responses of DC and macrophages to β-glucan-bearing particles pointed to changes in the pattern of expression of receptors and/or in the mechanisms of signal transduction. No major differences were observed in the expression of receptors in serum-differentiated macrophages in the presence and absence of LPS priming (Figure 6A). In contrast, DC showed a higher expression of dectin-1 and DC-SIGN. Serum-differentiated macrophages differed from M-CSF differentiated macrophages by the presence of a lower number of fusiform cells (Fig. 6A). Since dectin-1 shows several isoforms, some of them with deletions in the CRD encoding region and in the transmembrane and stalk regions [5], RT-PCR assays were conducted to address the expression of these isoforms. Day-one monocytes only expressed the mRNA encoding dectin-1 D isoform, which is characterized by the deletion of both the stalk region and a portion of the carbohydrate recognition domain (CRD) (Figure 6B). Incubation with serum and M-CSF induced dectin-1 B and to a lower extent dectin-1 A, whereas dectin-1 B was the predominant isoform in DC. To quantify these changes, real-time RT-PCR was carried with primers spanning the extracellular and intracellular portions of dectin-1 and with a reverse primer designed from the exon skipped in dectin-1 D to assay dectin-1 A and B isoforms. Differentiation with M-CSF enhanced the expression of dectin-1 A and B isoforms as compared to the levels observed in serum-differentiated macrophages (Figure 6C). Analysis of the mRNA encoding other receptors showed that DC-SIGN is the receptor displaying the highest expression increase (Figure 6C, right panels), thus agreeing with the reported effect of M-CSF on regulatory macrophages [30]. These results indicate that differentiation of monocytes into macrophages has a prominent effect on the expression of dectin-1 and DC-SIGN, and that these changes can explain the mechanism whereby the response to β-glucans and α-mannans vary along the differentiation process.

### Effect of the Deletion of dectin-1

**Figure 7:**
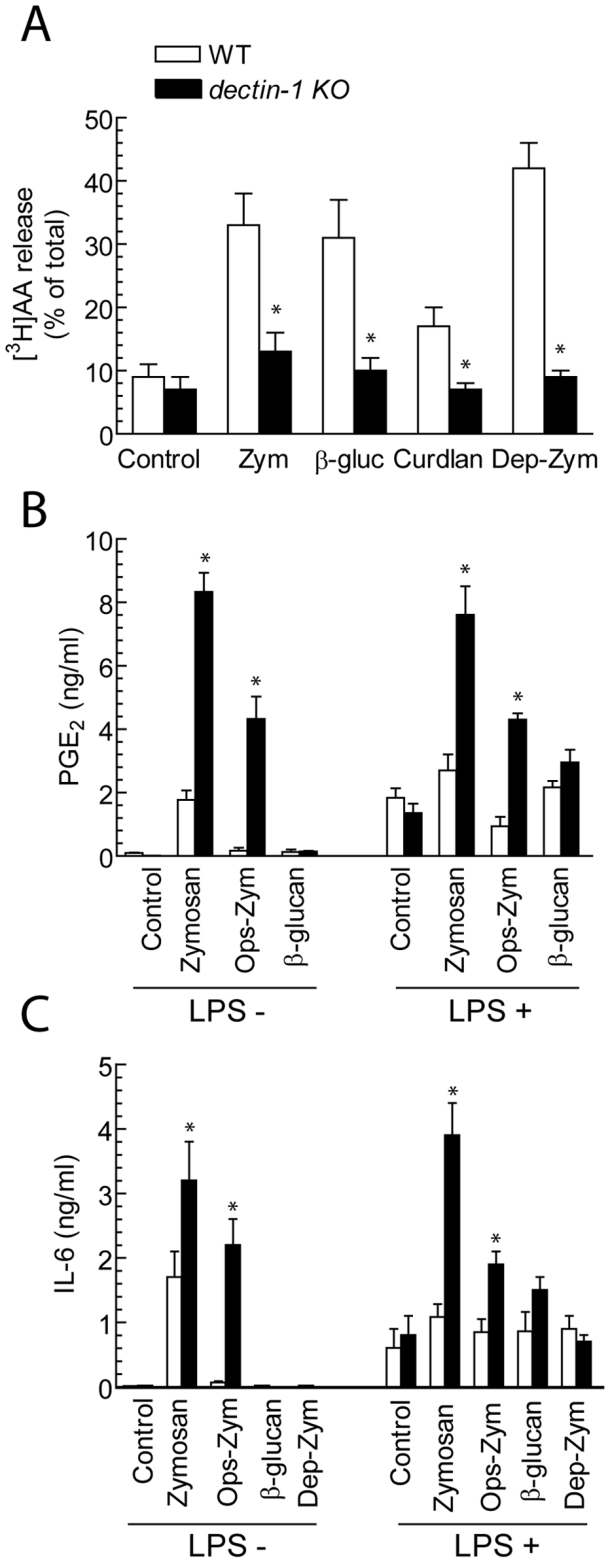
Effect of *clec7a* deletion on the response to β-glucans. (A) BMDM from WT and *dectin-1^−/−^* mice were used for the release of [^3^H]AA after incubation for 1 hour with the indicated additions at the concentration of 1 mg/ml. (B) The production of PGE_2_ was assayed after 24 hour in BMDM preincubated for 3 hours with 10 ng/ml LPS or left untreated. (C) Production of IL-6 assayed 24 hours after the addition of the stimuli. Results indicate mean ± S.D. of 4 to 6 independent experiments. *Indicates *p*<0.05 as compared to WT mice.

Experiments in mouse bone marrow-derived macrophages (BMDM) were carried out to address the receptors involved in the response to β-glucans. *dectin-1^−/−^* mice did not release [^3^H]AA in response to a set of different β-glucan-bearing stimuli (Figure 7A). However, zymosan was a potent stimulus for the release of PGE_2_, whereas pure β-glucan was ineffective (Figure 7B). Notably, the production of PGE_2_ elicited by zymosan was enhanced in *dectin-1^−/−^* mice as compared to the WT. A similar pattern of response was observed for IL-6 (Figure 7C). Altogether, these results indicate the central role of dectin-1 in the induction of [^3^H]AA release by β-glucans.

### Adaptor Proteins and Tyrosine Phosphorylation Reactions

**Figure 8:**
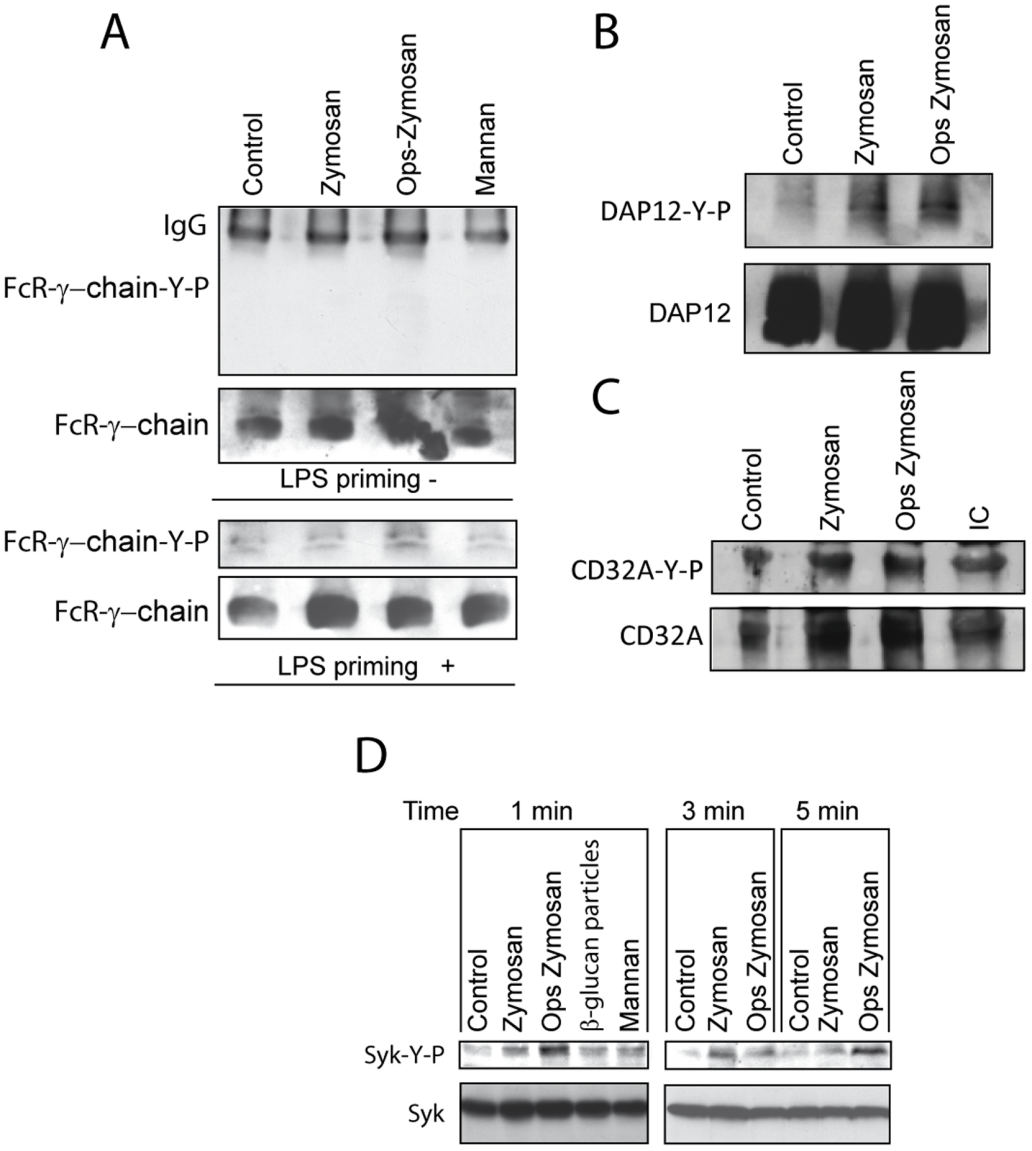
Immunoprecipitation and assay of tyrosine phosphorylation of FcR γ-chain, DAP12, and CD32A. Macrophages were differentiated with serum and incubated overnight with medium containing BSA. At the end of this period, they were treated for 1 min with the stimuli. Cell lysates were used for the immunoprecipitation of (A) FcR γ-chain, (B) DAP12, and (C) CD32A. After separation of the proteins in SDS/PAGE and transference to nitrocellulose membranes, the different proteins were immunodetected. (D) The tyrosine phosphorylation of Syk was assayed with phosphospecific Ab. These are representative blots of two experiments with similar results.

Since opsonisation of zymosan enhances productive binding to the β_2_-integrin CR3 and tyrosine phosphorylation of ITAM-containing adaptors leads to Syk activation following integrin engagement [19], we addressed whether zymosan induced the phosphorylation of DAP12and FcR γ-chain. Neither zymosan nor opsonized zymosan induced tyrosine phosphorylation of FcR γ-chain (Figure 8A). In contrast, they increased tyrosine phosphorylation of DAP12 and to a lower extent of CD32 (Figure 8B and C). Consistent with the downstream activation of Syk following phosphorylation of adaptor proteins, phosphorylation of Syk Y525/526 was detected as early as 1 min after stimulation (Figure 8D). Taken collectively, these data indicate that in addition to the canonical route involving dectin-1, zymosan particles may activate Syk by a mechanism involving the recruitment of DAP12. In keeping with the involvement of CD32A in CR3 signaling [31], a CD32A blocking Ab significantly inhibited [^3^H]AA release in response to opsonized β-glucan particles (Figure 2B).

### Activation of the NF-κB Route

**Figure 9:**
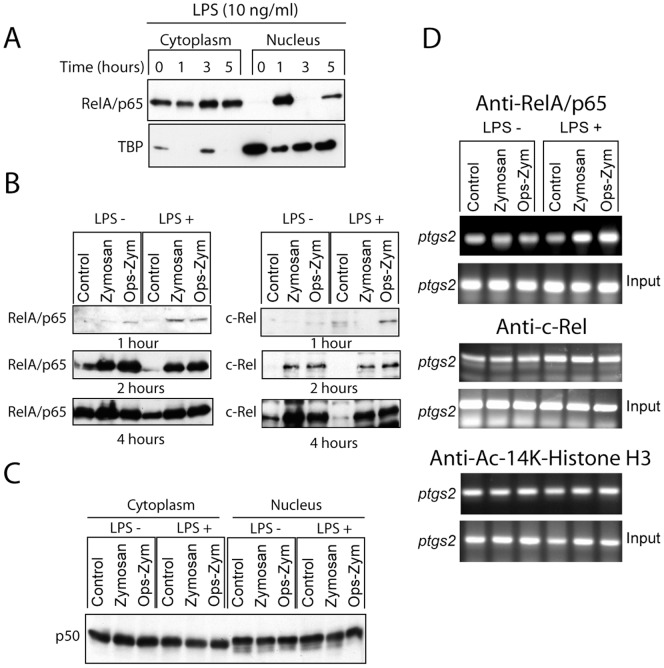
Activation of NF-κB proteins by LPS and zymosan. (A) Macrophages were treated for up to 5 h with 10 ng/ml LPS and RelA/p65 was assayed in the cytoplasm and nucleus. (B) Macrophages were primed for 3 h with LPS and then stimulated for different times. (B–C) The nuclear fractions were used for the assay of RelA/p65, c-Rel, and p50. (D) Binding of RelA/p65 and c-Rel, and histone H3 acetylation of the *ptgs2* promoter were assayed in the presence and absence of LPS priming 2 h after stimulation. These are representative experiments of at least two showing identical results.

Since the NF-κB family of transcription factors plays a major role in the transcriptional regulation of COX-2, we addressed the involvement of this route. Priming with LPS induced a rapid activation of NF-κB as judged from the nuclear translocation of RelA/p65. This was followed by their disappearance from nuclear fractions and the reappearance of the protein at 5 hours, which can be explained most likely through an indirect mechanism involving the formation of secondary mediators (Figure 9A). When zymosan was used as the stimulus, nuclear translocation of RelA/p65 and c-Rel was observed 2 and 4 hours after the addition of the stimuli, thus suggesting a more delayed time-pattern of response than that elicited by LPS (Figure 9B). In contrast, p50 was detected in the nuclear fractions both upon stimulation and under resting conditions (Figure 9C). Notably, zymosan induced the binding of RelA/p65 to the *ptgs2* promoter in LPS-primed cells two hours after addition of the stimuli, but it did not increase c-Rel binding over the level detected in LPS primed cells (Figure 9D). We did not observe any significant change of the level of histone H3 acetylation in the *ptgs2* promoter following LPS priming (Figure 9D lower panel). Since NF-κB activation and COX-2 induction have been reported to occur after crystal particle uptake [25], [32] with a time-course similar to that observed with zymosan particles by a mechanism involving the release of cathepsin B from the phagolysosome [20], we addressed the effect of cytochalasin D, an alkaloid that inhibits phagocytosis, the cathepsin B inhibitor CA-074, and the lysosomal stabilizing agent poly-2-vinylpyridine N-oxide (PVNO) on PGE_2_ production. As shown in Figure 3, cytochalasin D and CA-074 inhibited the response to both zymosan and opsonized zymosan, whereas the inhibition by PVNO did not reach statistical significance. Taken together, these results suggest that particle uptake and cathepsin B can be involved in the delayed production of PGE_2_ elicited by zymosan. Further evidence of the parallelism between the effect of zymosan and crystal particles was the observation that latex particles also induced COX-2 expression in serum differentiated macrophages (Figure 4F), even taking into account the differences in the load of protein in the different lanes.

## Discussion

These data show that the response of macrophages to β-glucans depends on their state of differentiation and the presence of LPS and/or cytokines in the microenvironment. Whereas several studies have addressed this issue in mouse macrophages, to the best of our knowledge this is the first systematic study on human macrophages. Our findings can be summarized in three distinct patterns: i) a pattern consistent with phagosomal processing, ii) a pattern induced by priming with LPS, and iii) a pattern elicited by M-CSF, the hallmark of which is an increased expression of dectin-1 B isoform.

The first pattern is reminiscent of both the response to crystals and the increased access to intracellular pattern receptors elicited by phagosomal processing [33]. Notably, a recent study has disclosed that dectin-1 dependent response to *Aspergillus* conidia occurs when the particles are located in acidified phagolysosomes, thereby stressing the importance of β-glucan signalling from the phagolysosome [34]. Our findings show an early activation of Syk and a more delayed activation of the NF-κB route that leads to the induction of COX-2, IL-6, and IL-23. The production of PGE_2_ is sensitive to cathepsin B inhibition and to the blockade of phagocytosis. These findings agree with the activation of cathepsin B by β-glucans [35] and with the inhibitory effect of cytochalasin D on cytokine production in DC stimulated with particulate β-glucans reported by Rosas et al. [9], who concluded that inhibition of cytoskeletal assembly by cytochalasins impides the contact with particles. A study addressing caspase recruitment domain-containing protein (CARD)9 by dectin-1 signaling showed that it is possible to distinguish signals downstream Syk such as activation of p38 MAPK, which can occur in the absence of cytokine production, and signals dependent on Syk/CARD9/NF-κB associated with cytokine production [10]. In other words, the dectin-1 route can promote CARD9 phagosomal translocation and enhance TLR-induced cytokine production even though dectin-1 signaling is insufficient to drive cytokine production. As it has been reported that phagosomal degradation increases TLR access to ligands [33], it seems likely that a similar mechanism may be operative following zymosan phagocytosis, thus agreeing with the recognition of *Aspergillus* conidia by dectin-1 in acidified phagolysosomes [34].

By using BMDM from *dectin-1* ^−/−^ mice, we observed that dectin-1 is necessary for AA release by β-glucans and dispensable for the cytokine response. The absence of a delayed production of PGE_2_ by pure β-glucan in WT mice in spite of the occurrence of AA release, can be explained by the absence of COX-2 induction. Unexpectedly, PGE_2_ and IL-6 production were enhanced in the *dectin-1^−/−^* mice, thus suggesting that in the absence of dectin-1, zymosan binding to receptors that activate κB-dependent transcription such as TLR2 might be enhanced. A similar mechanism could explain the lower production of IL-6 and IL-23 in human macrophages differentiated with M-CSF A plausible explanation for the prominent IL-6 response to zymosan particles is that the transcriptional regulation of *il6* involves cooperation of NF-κB and cyclic AMP response element binding protein (CREB) to recruit CREB-binding protein (CBP) at the *il6* promoter, thus enabling synergistic gene activation [36]. In keeping with these data, the time course of the activation of these factors seems optimal given that NF-κB activation by zymosan shows a delayed time-course that seems coincidental with the generation of PGE_2_ and the ensuing activation of the E prostanoid receptor/protein kinase A/cyclic AMP/CREB system. This pattern of cooperation differs from other systems where CREB and RelA/p65 bind to different promoters and may compete for the coactivator CBP [37]. The predominant production of IL-23 could be explained by the ability of zymosan to blunt *il12a* transcription [38].

Macrophages exposed to a low concentration of LPS show an early induction of COX-2 that may be the consequence of the first wave of the activation of NF-κB by the TLR4 route. This agrees with current views on *ptgs2* regulation, in that it is a primary response gene with CpG islands and constitutive histone acetylation that allows a transcriptionally permissive state [39]. Although zymosan clearly enhanced the production of PGE_2_ and the activation of NF-κB over that observed upon LPS exposure, the immunodetection of COX-2 protein did not show a parallel increase, which can be explained by the instability of COX-2 protein [40]. In this connection, COX-2 induction has always been assayed in the presence of load controls. Although the expression of the putative housekeeping gene might show variations among different lanes due to either technical mistakes or inconsistent behaviour of the housekeeping gene. For instance, the β-actin load in the rightmost lane in Figure 4F. It seems likely that the induction of COX-2 has been underestimated rather than overestimated, therefore not affecting the overall significance of the results. The enhancement by zymosan of the effect of a priming dose of LPS was also shown at the *ptgs2* promoter as judged from an increased binding of RelA/p65. An effect related to an increased acetylation of the *ptgs2* promoter seems unlikely, in view of the steady levels of histone H3 acetylation. As to the effect of IFNγ, we did not observe an enhanced expression of COX-2 protein. This agrees with the reported effect of IFNγ on microsomal PGE synthase expression, since co-stimulation of TNFα with IFNγ reduces COX-2 protein expression, but enhances PGE_2_ biosynthesis through an increased expression of PGE synthase [41].

M-CSF enhanced the expression of the isoforms of dectin-1 most usually associated with productive binding, specially dectin-1 B, and increased AA release and PGE_2_ production in the absence of an increased expression of cPLA_2_ and COX-2. The enhanced production of LTB_4_ observed upon M-CSF treatment agrees with a rapid formation by 5-lipoxygenase, the activity of which is regulated by AA disposal. Most studies so far conducted have been carried out in GM-CSF treated cells [14], [42] and have disclosed an increased expression of receptors involved in the recognition of α-mannan and β-glucan moieties rather than an increased expression of cPLA_2_. In contrast, M-CSF has been reported to down-regulate the production of cytokines by mouse macrophages [9], [10]. Given that M-CSF increases the expression of the dectin-1 B isoform as well as the expression of the α-mannan binding receptor DC-SIGN, our findings suggest that similar to the effect of GM-CSF, the main effect of M-CSF on AA metabolism in human macrophages might be exerted on the expression of receptors rather than on the induction of enzymes.

**Figure 10:**
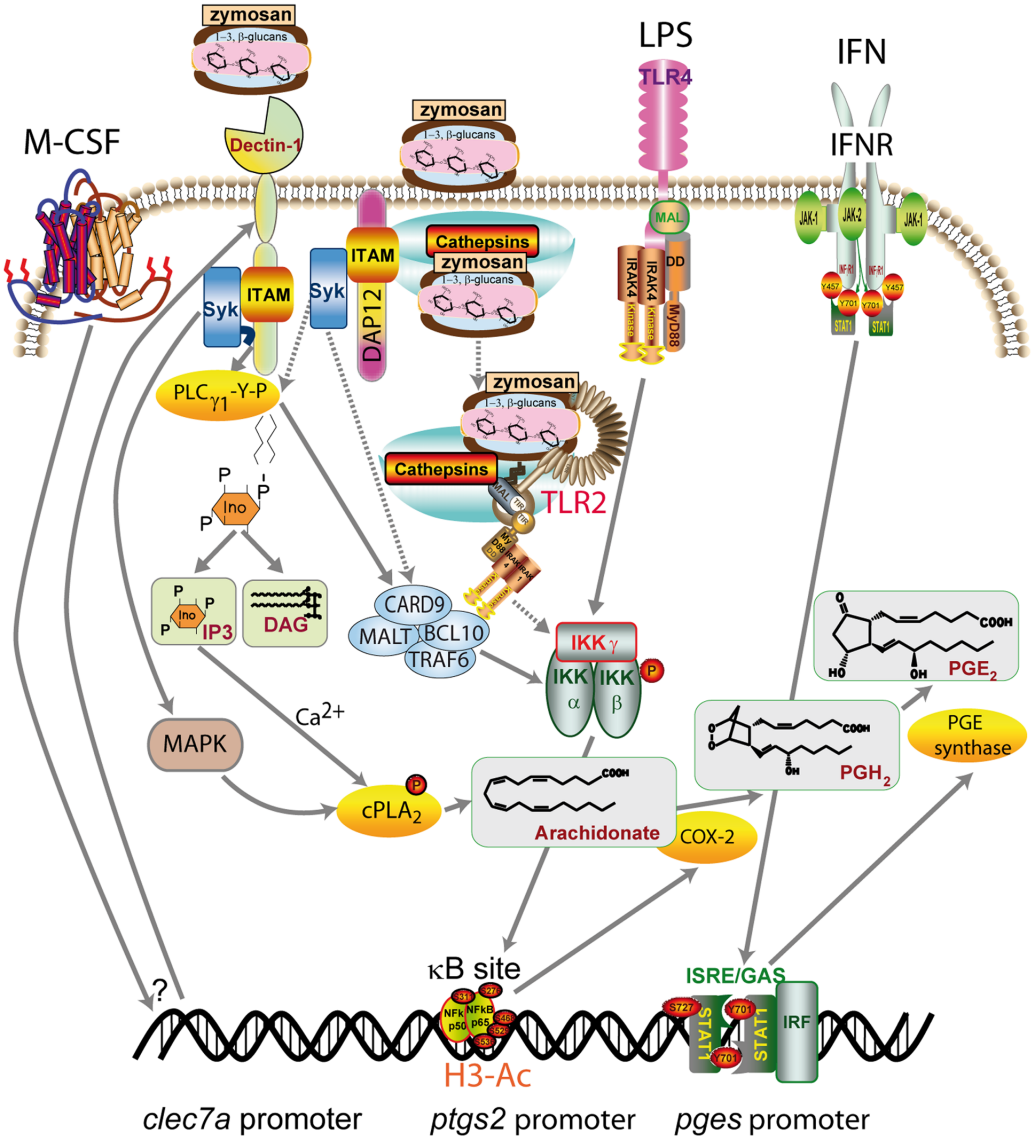
Proposed mechanisms involved in zymosan uptake and signaling in human macrophages. Zymosan particles can activate Syk via dectin-1 engagement and through the adaptor protein DAP12. After internalization into phagosomes, TLR2 recognition and cathepsin B leakage may occur. In the presence of M-CSF, the expression of dectin-1 B isoform is increased and allows for an enhanced receptor-dependent binding. If the LPS/TLR4 cascade is activated, an additional, concomitant mechanism of NF-κB activation will be triggered. cPLA_2_ is activated by dectin-1/Syk-dependent mechanisms involving MAPK-dependent Ser-505 phosphorylation and Ca^2+^-dependent membrane translocation, whereas the induction of COX-2 depends mainly on κB-dependent transcriptional regulation. IFNγ signaling leads to the activation of the promoter of the inducible microsomal isoform of prostaglandin E synthase (*pges*) by an IFN-stimulated response element (ISRE)-dependent or GAS (interferon-γ activated sequence)-dependent mechanism. The dotted lines indicate the steps associated with phagocytic cargo processing. Ac-H3 indicates acetylated histone H3.

The role of adaptor proteins should be analyzed in the context of their role in integrin signaling [19] and fungal uptake [31]. We have detected phosphorylation of DAP12 and CD32A, but we did not observe phosphorylation of Fc receptor γ-chain. Unlike DAP12 tyrosine phosphorylation, CD32A phosphorylation did not increase upon zymosan challenge, what agrees with the notion that basal ITAM phosphorylation is commonly observed in macrophages in the absence of ligand binding and that low-level ITAM-mediated signaling might provide functionally relevant signals [23], [43]. Since tyrosine phosphorylation of DAP12 followed zymosan stimulation, it seems likely that receptor-independent, direct membrane binding signaling might utilize DAP12 to recruit Syk [23]. A schematic diagram of our findings is shown (Figure 10). Taken collectively, our data show the existence of distinct patterns of response of human macrophages to β-glucan containing particles, the scope of which varies from a limited and delayed response mimicking signaling from the phagosomes to a synergistic response triggered by priming with LPS and cytokines.

## Supporting Information

Figure S1 **Mass spectrometric characterization of the eicosanoids released by macrophages.** The supernatants of 4 ml of culture medium corresponding to ∼2.10^6^ macrophages differentiated in the absence (A) and presence of M-CSF (B), primed with 10 ng/ml LPS and stimulated with zymosan were extracted and analyzed as explained in Materials S1. The upper panels in (A) and (B) show a display arranged to show maximal intensity of the recording as a function of free AA (m/z 303.233). Middle and lower panels have been adapted to show maximal intensity for LTB_4_ (m/z 335.222) and PGE_2_/PGD_2_ (m/z 351.217), respectively.
(TIF)

Materials S1 **Eicosanoid measurements by reversed phase ultra performance liquid chromatography (UPLC) and electrospray-quadrupole-time-of-flight-mass spectrometry.**
(DOC)
