# Supplementary figures and images for: The Response of Human Macrophages to β-Glucans Depends on the Inflammatory Milieu
Source: PLoS One. 2013 Apr 24;8(4):e62016. doi: 10.1371/journal.pone.0062016 (PMC3634770; doi:10.1371/journal.pone.0062016)

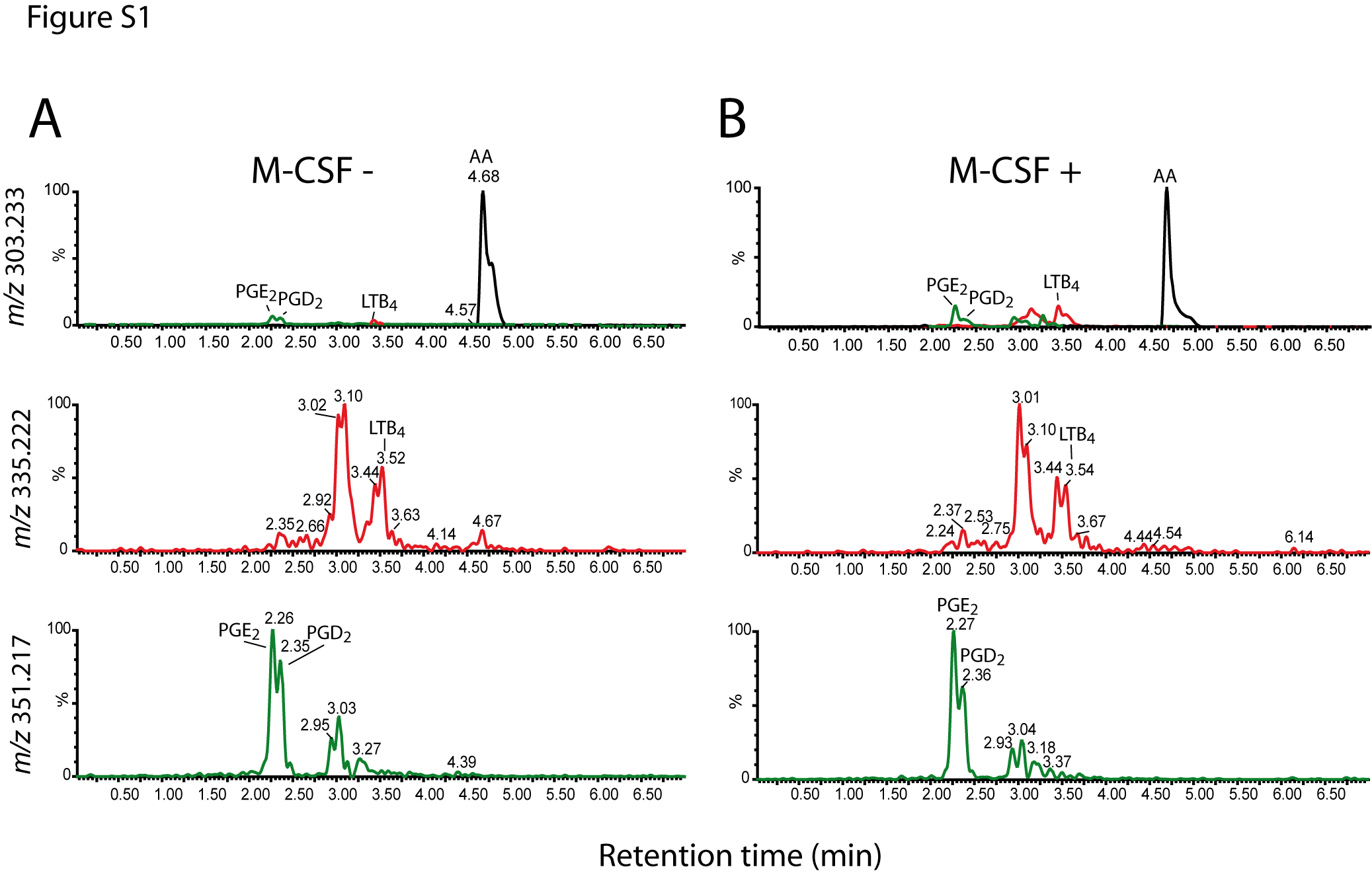

Supplement: Figure S1 — Mass spectrometric characterization of the eicosanoids released by macrophages. The supernatants of 4 ml of culture medium corresponding to ∼2.106 macrophages differentiated in the absence (A) and presence of M-CSF (B), primed with 10 ng/ml LPS and stimulated with zymosan were extracted and analyzed as explained in Materials S1. The upper panels in (A) and (B) show a display arranged to show maximal intensity of the recording as a function of free AA (m/z 303.233). Middle and lower panels have been adapted to show maximal intensity for LTB4 (m/z 335.222) and PGE2/PGD2 (m/z 351.217), respectively. (TIF) [file pone.0062016.s001.tif]
