# Supplementary material for: The Response of Human Macrophages to β-Glucans Depends on the Inflammatory Milieu
Source: PLoS One. 2013 Apr 24;8(4):e62016. doi: 10.1371/journal.pone.0062016 (PMC3634770; doi:10.1371/journal.pone.0062016)
Supplement: Materials S1 — Eicosanoid measurements by reversed phase ultra performance liquid chromatography (UPLC) and electrospray-quadrupole-time-of-flight-mass spectrometry. (DOC) [file pone.0062016.s002.doc]

Eicosanoids were eluted in methanol using Strata^TM^ C-18E SPE tubes (Phenomenex) and evaporated to dryness under N_2_. The solid residue was solubilized in 40 μl UPLC solvent A (8.3 mM acetic acid buffered to pH 5.7 with ammonium hydroxide) plus 20 μl UPLC solvent B (acetonitrile/methanol 65:35, v/v). A 7.5 μl aliquot of each sample was injected into the chromatographic system. The chromatographic separation was conducted in an Acquity™ UPLC System (Waters, Manchester, UK) equipped with an Acquity UPLC® BEH C18, 1.7 μm, 2.1×50 mm column (Waters). The chromatographic column was directly interfaced into the electrospray source of a mass spectrometer (SYNAPT HDMS G2, Waters). Mass spectrometric analysis was performed in negative ion mode using a MS^E^ method that allows simultaneous detection of analytes through a low energy function (full scan) and a high energy function (collision energy) with ion partial fragmentation. The mass spectrometer was operated in sensitivity mode with 5 ppm threshold for maximum RMS residual mass. Sodium formate was used for calibration and leucine-enkephaline (*m/z* 554.2615) for reference of mass accuracy. For quantification, external standards of PGE_2_, PGD_2_, LTB_4_, and AA were used to draw a linear regression of chromatographic peak area to compound concentration. All compounds were identified by injecting authentic standards and by exact mass along with fragmentation pattern as observed in the high energy function. The parent ions of *m/z* 351.217, *m/z* 335.222, and *m/z* 303.233, as obtained in the low energy function, were used for quantification of PGE_2_, LTB_4_, and AA, respectively, while the fragment ion of *m/z* 315.196 was used for quantification of PGD_2_.
